# Function identification of miR482b, a negative regulator during tomato resistance to *Phytophthora infestans*

**DOI:** 10.1038/s41438-018-0017-2

**Published:** 2018-03-01

**Authors:** Ning Jiang, Jun Meng, Jun Cui, Guangxin Sun, Yushi Luan

**Affiliations:** 1 School of Life Science and Biotechnology, Dalian University of Technology, Dalian, 116024 China; 2 School of Computer Science and Technology, Dalian University of Technology, Dalian, 116024 China

## Abstract

Tomato is an important horticultural and economic crop cultivated worldwide. As *Phytophthora infestans* becomes a huge threat to tomato production, it is necessary to study the resistance mechanisms of tomato against *P. infestans*. Our previous research has found that miR482 might be involved in tomato–*P*. *infestans* interaction. In this study, miR482b precursor was cloned from *Solanum pimpinellifolium* “L3708” and miR482b was shown to decrease in abundance in tomato following *P*. *infestans* infection. Compared to wild-type tomato plants, tomato plants that overexpressed miR482b displayed more serious disease symptoms after *P*. *infestans* infection, with more necrotic cells, longer lesion diameters, and increased *P*. *infestans* abundance. Meanwhile, silencing of miR482b was performed by short tandem target mimic (STTM), resulting in enhancement of tomato resistance to *P*. *infestans*. Using miRNA and degradome data sets, *NBS–LRR* disease-resistance genes targeted by miR482b were validated. Negative correlation between the expression of miR482b and its target genes was found in all *miR482b*-overexpressing and -silencing tomato plants. Our results provide insight into tomato miR482b involved in the response to *P*. *infestans* infection, and demonstrate that miR482b–*NBS–LRR* is an important component in the network of tomato–*P*. *infestans* interaction.

## Late blight resistance genes identified in tomato

Researchers in China have identified a genetic pathway in tomato involved in resistance to the late blight pathogen, *Phytophthora infestans*. A team at Dalian University of Technology led by Yushi Luan engineered tomatoes to express more or less miR482b and then infected them with late blight. Symptoms were more severe in plants with excess miR482b and attenuated in those with reduced miR482b. The researchers also predicted the targets of miR482b and found 30 defense-related genes known as NBS-LRRs. Sequencing revealed that four of these genes were degraded in tomatoes infected with late blight. In addition, the expression of all four decreased when miR482b was strongly expressed and increased when it was silenced. These findings identify miR482b-NSS-LRR as an important pathway in tomato’s response to *P*. *infestans* which may be a valuable target for molecule breeding.

## Introduction

Tomato, as an important horticultural and economic crop cultivated worldwide, always suffers from the aggression of various pathogens^1–3^. *Phytophthora infestans*, the agent that causes late blight, has become one of the most devastated pathogens^4^ and can cause great losses in tomato production^5^. For example, in Inner Mongolia of China and the USA, late blight caused the loss of tomato production in early 2000s^6,7^. Up to now, the molecular mechanisms of tomato–*P*. *infestans* interaction are beyond understanding and the method for controlling tomato late blight is still not effective enough. It is necessary to study tomato resistance mechanisms against *P*. *infestans* and identify the key resistance genes which are used in disease-resistance breeding of tomato transgene.

MicroRNA (miRNA), a class of about 22 nt endogenous small noncoding RNA, have been discovered to play an important role in regulating endogenous gene expressions in plants^8,9^. MiRNAs attack their targets by sequence-specific binding to the targeting sites on the gene transcripts, leading to either target mRNA degradation or translational repression mediated by the miRNA-associated proteins^10^. Up to now, the major methods to study miRNA functions are to generate and analyze the transgenic lines overexpressing miRNAs^11,12^. For example, in our previous studies, overexpression of miR172 in tomato enhanced its susceptibility to *P*. *infestans*^13^. Meanwhile, the tomato resistance to *P*. *infestans* was reduced by the overexpression of miR1918^14^. However, these approaches are insufficient to fully understand miRNA functions. Short tandem target mimic (STTM), which was invented based on an endogenous mechanism that modulates miR399 activity in *Arabidopsis thaliana*^15^, is a newly developed approach to explore miRNA functions by blocking miRNAs in plants^16^. In previous studies, STTM had been used to silence miR165/166, miR160, and miR159 in *A*. *thaliana*, cotton, soybean, tobacco and these miRNAs were found functioning as regulators of plant growth and development^17–21^. In tomato, blocking miR858 by overexpressing STTM858 resulted in high accumulation of anthocyanins, which demonstrated miR858 could negatively control the anthocyanin accumulation of tomato^22^.

Many miRNAs have been demonstrated to play important roles in stress responses. A number of plant miRNAs, such as miR159, miR160, miR166, miR169, miR172, and miR396, are involved in the response to drought, water deficit, and salt stresses^23,24^. More and more evidence have indicated that plant miRNAs also play an important role in plant–pathogen interaction^25,26^. For example, miR393 and miR472 can contribute to pathogen-associated molecular pattern-triggered immunity (PTI) in plants^27,28^. Other miRNAs, such as miR482, miR6019, miR6020, miR6022, miR5300, miR1507, and others, are thought to suppress nucleotide-binding site and leucine-rich repeat (*NBS*–*LRR*) defense genes, which play critical roles in effector-triggered immunity (ETI), also a crucial component of plant immune system^29–33^.

MiR482, a class of miRNAs found in various plants, acts in plant–pathogen interaction^34^. Previous studies have shown, miR482 is involved in the process of tomato, cotton, soybean, and peanut interacting with Potato spindle tuber viroid, Cucumber mosaic virus, *Verticillium dahliae*, soybean cyst nematode, and *Ralstonia solanacearum*^35–39^. MiR482 regulates defense mechanisms of plant via targeting conserved sequences encoding the P-loop of NBS–LRR resistance proteins^38^. In cotton, ~12% of cotton *NBS–LRR* genes were predicted targets by gra-miR482 family^40^. In Solanaceae, miR482 was also found targeted to *NBS–LRR* genes^30,31,34^. *NBS–LRR* genes were the most represented gene family of resistance (R) genes^41^. They can enhance plant resistance to various pathogens, such as nematodes, *Magnaporthe oryzae*, *Xanthomonas oryzae*, tomato mosaic virus (ToMV), and *Pseudomonas syringae*^42–46^. Previous study in potato found the increased susceptibility of miR482e-overexpressing plants to *V*. *dahliae* infection could be explained by the enhancement of miR482e-mediated silencing on *NBS–LRR* disease-resistance genes^31^.

Using high-throughput sequencing and homology-based computational research, we have previously identified a number of tomato miRNAs involved in tomato–*P*. *infestans* interaction including miR482, miR172, miR6024, miR6026, miR6027, etc.^13,25,47,48^. Our previous work showed that only miR482b was identified in tomato, *Solanum pimpinellifolium* “L3708” (L3708) inoculated with *P*. *infestans* and its transcripts per million clean tags was lower than wild-type (WT) tomato after analysis of the miRNA-Seq data. However, whether miR482b affected tomato resistance to *P*. *infestans* has not been determined. In this study, we analyzed the expression patterns of miR482b in tomato L3708 after *P*. *infestans* treatment. Then the function of miR482b was explored by overexpressing and silencing approach and its target genes were also identified. These studies will benefit not only understanding of the molecular mechanisms of tomato–*P*. *infestans* interaction but also future molecular breeding.

## Materials and methods

### Plant material collection and *P*. *infestans* inoculation

Tomato L3708 was grown in a greenhouse at 25 ± 3 °C under 16 h of light per day. *P*. *infestans* strain “P12103” was cultured in oat medium in the dark at 20 °C. Tomato plants at the five to six leaf stage were inoculated with *P*. *infestans* spores suspension (10^6^ zoospores mL^−1^) and placed at 20 ± 1 °C in a 100% relative humidity environment without light. Tomato plants treated with sterile water were used as controls, and they were kept under the same conditions. Leaf samples were collected at 0, 1, 2, 3, and 4 days post inoculation (dpi). All samples were quickly frozen in liquid nitrogen and stored at −80 °C until DNA and RNA isolation.

### Cloning of pre-miR482b

MiR482b precursor (pre-miR482b) was cloned using tomato DNA as a template. The primers (*miR482bF* and *miR482bR*; Table S2) were designed according to the tomato genome sequences using the Primer Premier 5 software. The PCR product was cloned into T-Vector pMD 19 (Takara, Dalian, China), and the identity of the sequence was verified by DNA sequencing. Multiple nucleic acid sequence alignments were performed with ClustalX2.

### Construction of silencing and overexpressing plasmid of miR482b and their transient overexpression in tomato

Silencing of miR482b with STTM was performed. The structure of STTM482b was design based on the description of Yan et al.^17^. It contained two copies of imperfect miR482b binding sites (22 nucleotides) linked with a 48 nt linker. A bulge contain three additional nucleotides (CTA) was designed around positions 10 to 11 of the miRNA to trap miR482b without being cleaved by it. The pre-miR482b and STTM482b inserts were then subcloned into *Bam*HI–*Sac*I cut pBI121 to generate the recombinant overexpressing plasmids pBI121-miR482b and silencing plasmid pBI121-STTM482b, in which the expression of miR482b and STTM482b are controlled by the strong constitutive CaMV35S promoter. Freeze-thaw method was used to introduce pBI121-miR482b and pBI121-STTM482b into *Agrobacterium tumefaciens* strain “GV3101”.

A sample from *A*. *tumefaciens* culture was centrifuged at 4000 × *g* for 10 min and the pellet was resuspended in infiltration medium (10 mM MgCl_2_, 10 mM MES and 20 μM acetosyringone; OD_600_ = 1.0)^49^. *A*. *tumefaciens* was introduced into tomato leaves by infiltration. *A*. *tumefaciens* with empty vector was used as a control. After 3 days, each infiltrated leaf region was inoculated with 20 μL of *P*. *infestans* (10^6^ zoospores mL^−1^). The lesions were observed at the 5th day and the length of the lesion diameter was calculated.

### Transformation and identification of transgenic tomato

Tomato transformation was performed based on the method of Li et al.^50^. The seeds of tomato *S*. *lycopersicum* “Zaofen No. 2” were cultured in 1/2 Murashige and Skoog (MS) medium at 25 ± 3 °C under 16 h of light per day after surface sterilized with 75% (v/v) ethanol and 2.5% (v/v) sodium hypochlorite. Cotyledon excised from 1-week-old seedlings were used as explants. Transgenic tomato plants were generated by *A*. *tumefaciens*-mediated leaf disk method. Putative transgenic plants were selected on 1/2 MS medium with 200 mg L^−1^ carbenicillin and 50 mg L^−1^ kanamycin. After obtaining *T*_0_ kanamycin-resistant plants, the presence of the transgene in the regenerating plantlets was further confirmed using PCR with a pair of specific primers of *neomycin phosphoryltransferase II* (*nptII*) (Table S2). The abundance of miR482b in the selected positive overexpressing and silencing lines were examined by quantitative reverse transcription-polymerase chain reaction (qRT-PCR).

### Analysis of transgenic tomato resistance against *P*. *infestans*

The detached leaves of WT and transgenic tomato plants were inoculated with 10 μL of 10^6^ zoospore mL^−1^ zoospore suspension of *P*. *infestans* according to the method of Li et al.^50^. In whole-plant assays, a suspension of *P*. *infestans* zoospores (10^6^ zoospores mL^−1^) was also used to spray plants to run off. The inoculated leaves or whole plants were maintained at 20 ± 1 °C under high humidity condition for 24 h without light, and then placed in a greenhouse at 20 ± 1 °C under 16 h of light per day. Necrotic regions and disease indices were recorded at 5 dpi. UV photographs were also taken at 5 dpi. Images of the leaves were taken and the diameters of lesions were measured. Trypan blue staining assays used to detect dead cells of harvested leaves was also based on the method described by Li et al.^50^. In addition, the transcript level of *P*. *infestans Actin* gene was used to indicate *P*. *infestans* growth in plant by qRT-PCR^51^. Categorization of disease grades (DG) was performed as described by Li et al.^50^, and the disease index (DI) was calculated according to the following formula:

$${\mathrm{DI}}({\mathrm{\% }}) = \frac{{{\sum} {\left( {{\rm{DG}}_i \times n_i} \right)} \times 100}}{{n \times {\rm{DG}}_{i{\rm{max}}}}}$$

where DG_*i*_ is the value of DG, *n*_*i*_ is the number of plants in each DG, and *n* is the total number of plants. Each experiment was carried out at least three times.

The leaves were also collected to measure the content of malonaldehyde (MDA) according to previously described method^50^.

### Identification of target genes

The mature-miR482b sequence was input into psRNATarget (http://plantgrn.noble.org/psRNAtarget/) and WMD3 (http://wmd3.weigelworld.org/cgi-bin/webapp.cgi) to predict its target genes in tomato. Tomato transcripts (cDNA library, version 2.4/2.3) were used as data sets. The degradome sequencing library of tomato inoculated with *P*. *infestans* was used to verify the targets. CleaveLand software was used to find the cleavage site of the miR482b targets^52,53^. T-plot figures were produced based on R language^54^.

### qRT-PCR analysis

The abundance of miR482b in tomato was examined by qRT-PCR. The specific forward primer for miR482b was designed following the method described by Varkonyi-Gasic et al.^55^. Quantitative RT-PCR was performed using the SYBR PrimeScript miRNA RT-PCR kit (Takara, Dalian, China) with Corbett Rotor Gene 3000 qRT-PCR machine (Corbett Research, Mortlake, Vic., Australia). The primers for the target genes were designed following the manufacturer’s instructions for the SYBR Premix Ex Taq II kit (Takara, Dalian, China). The miR482b cleavage sites of target genes were ensured between the forward and reverse primers binding sites. Information on all primers are shown in Table S2. *Actin* was used as a reference control gene for the miRNA and target gene qRT-PCR analysis. Of the nine leaves sampled in each experiment, three leaves were pooled into one biological replicate, resulting in three biological replicates.

### Statistical analysis

All statistical analysis of data was performed with Origin 8.5, and all data were expressed as means ± SEs from three independent experiments. Based on http://abacus.bates.edu/~ganderso/biology/resources/writing/HTW_Guide_Reporting_Statistics_3-7-2011.pdf, the capital letters were used over the error bars. Letters shared in common between or among the groups would indicate no significant difference.

## Results

### Cloning, tissue-specific expression and expression patterns of tomato miR482b under *P*. *infestans* infection

The pre-miR482b was cloned from tomato L3708 based on tomato sRNAs available in the miRBase and tomato genome. The result of multiple nucleic acid sequence alignments showed that the sequence of the pre-miR482b and mature-miR482b were identical to the miR482b from cultivated tomato *S*. *lycopersicum* in miRbase (Figure S1).

**Fig. 1:**
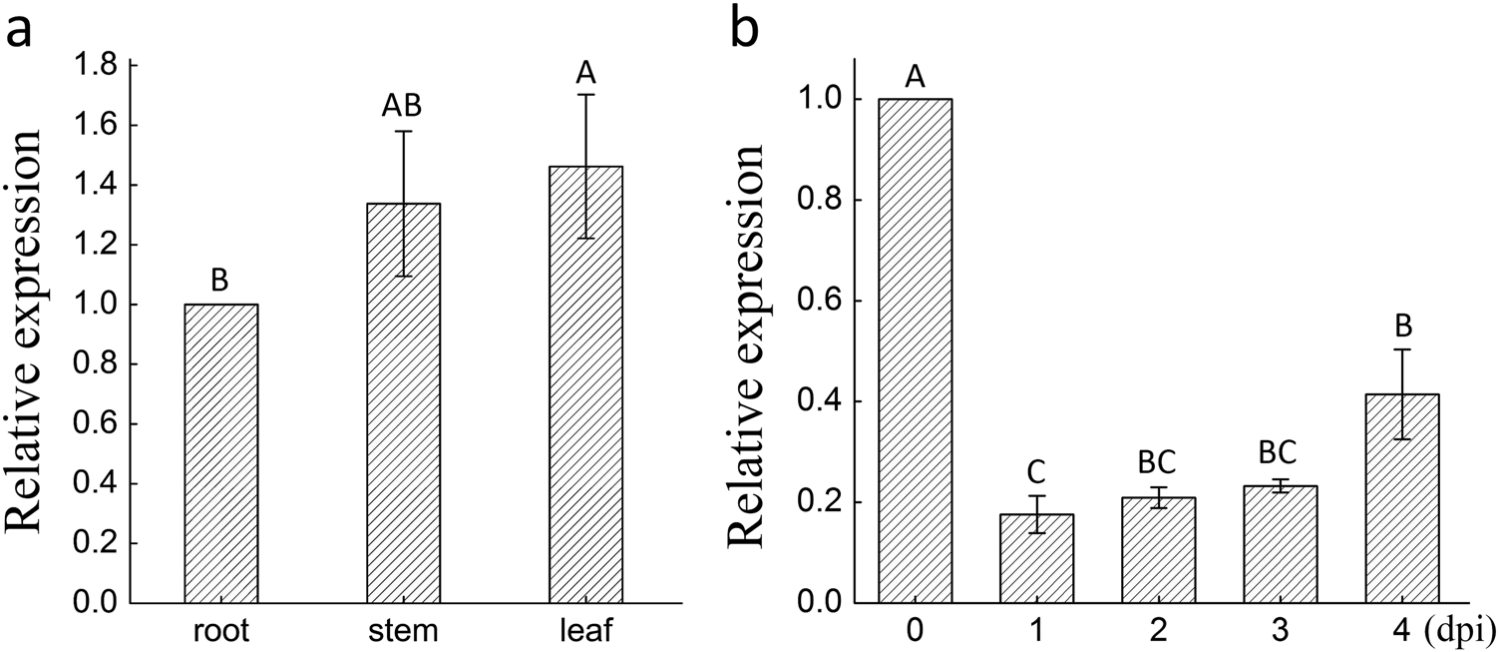
Quantitative real-time PCR analysis of the expression pattern of miR482b in different tissues and in response to *Phytophthora infestans*. **a** Tissue-specific expression of miR482b in tomato root, stem, and leaf. **b** Expression pattern of miR482b in tomato leaves at the indicated time after being sprayed with *P*. *infestans*. Data are the means ± SEs of three independent experiments. Letters indicate significant difference among samples, and letters shared in common between or among the groups indicate no significant difference at the *P* < 0.05 level

To analyze the distribution of miR482b within tomato L3708, qRT-PCR was used to measure the levels of miR482b transcript in the leaf, stem, and root of the plant. MiR482b was found in high abundance in leaf, whereas relatively low abundance was detected in root (Fig. 1a). To verify whether miR482b was involved in the response to *P*. *infestans* infection, the expression of miR482b in *P*. *infestans*-treated leaves at different dpi was measured. The result showed that after *P*. *infestans* treatment, the expression level of miR482b was lowest one day after the infection, but increased to a moderate level four days after the infection (Fig. 1b).

### Overexpression and silencing of miR482b by agrobacterium infiltration

**Fig. 2:**
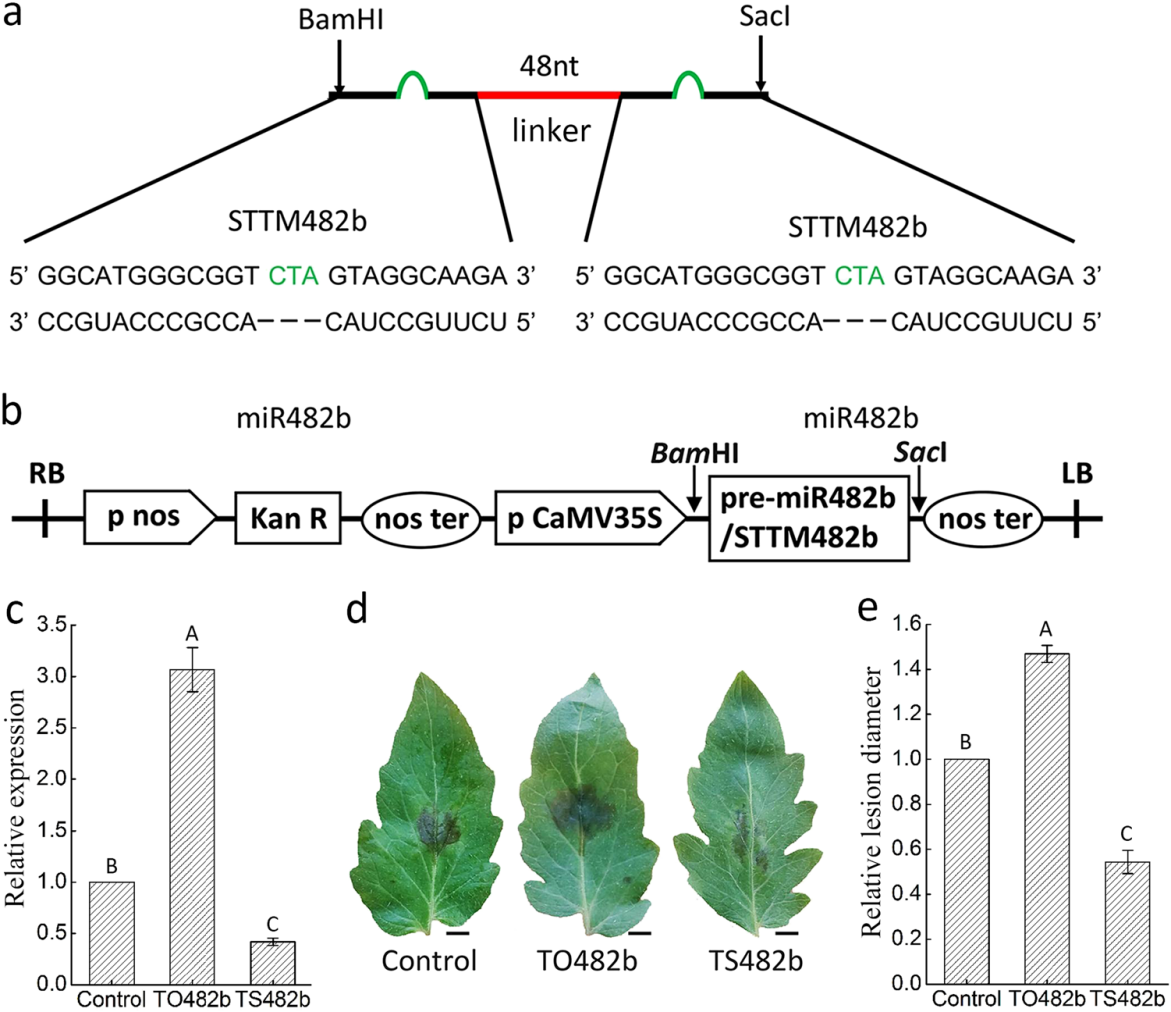
Transient overexpression and silencing of miR482b in tomato leaves. **a** Scheme of the base pairing pattern of STTM482b and miR482b. **b** Schematic diagram of gene cassette used for the overexpression and silencing of miR482b in tomato. **c** Relative quantities of miR482b in tomato leaves transiently overexpressing and silencing miR482b. **d** Disease symptoms of tomato leaves transiently overexpressing and silencing miR482b 5 days after inoculation with *P*. *infestans*. Control, transient overexpression of the empty vector; TO482b, transient overexpression of pBI121-miR482b; TS482b, transient silencing of pBI121-STTM482b. Scale bars = 0.5 cm. **e** The relative lesion diameter. Data are the means ± SEs of three independent experiments. Letters indicate significant difference among samples, and letters shared in common between or among the groups indicate no significant difference at the *P* < 0.05 level

STTM482b sequence was constructed to silence miR482b, which contained two copies of imperfect miR482b binding sites with a 48 nt linker and each cope had a cleavage-preventive bulge contained three additional nucleotides (CTA) (Fig. 2a). Then the overexpressing and silencing plasmids, pBI121-miR482b and pBI121-STTM482b were constructed (Fig. 2b) and introduced into tomato leaf by agrobacterium infiltration. The abundance of miR482b was significantly increased in tomato plants that transiently overexpressed miR482b (TO482b) and significantly decreased in tomato plants transiently silenced miR482b (TS482b) (Fig. 2c). Compared to control plants, TO482b tomatoes displayed more severe disease condition following infection of *P*. *infestans*, with significantly larger lesions in the leaves, while TS482b tomato showed less severe disease condition after *P*. *infestans* infection (Fig. 2d, e). These results suggested that miR482b may have weakened the resistance of tomato against *P*. *infestans*.

### Identification of transgenic plants with high and low miR482b expression levels

**Fig. 3:**
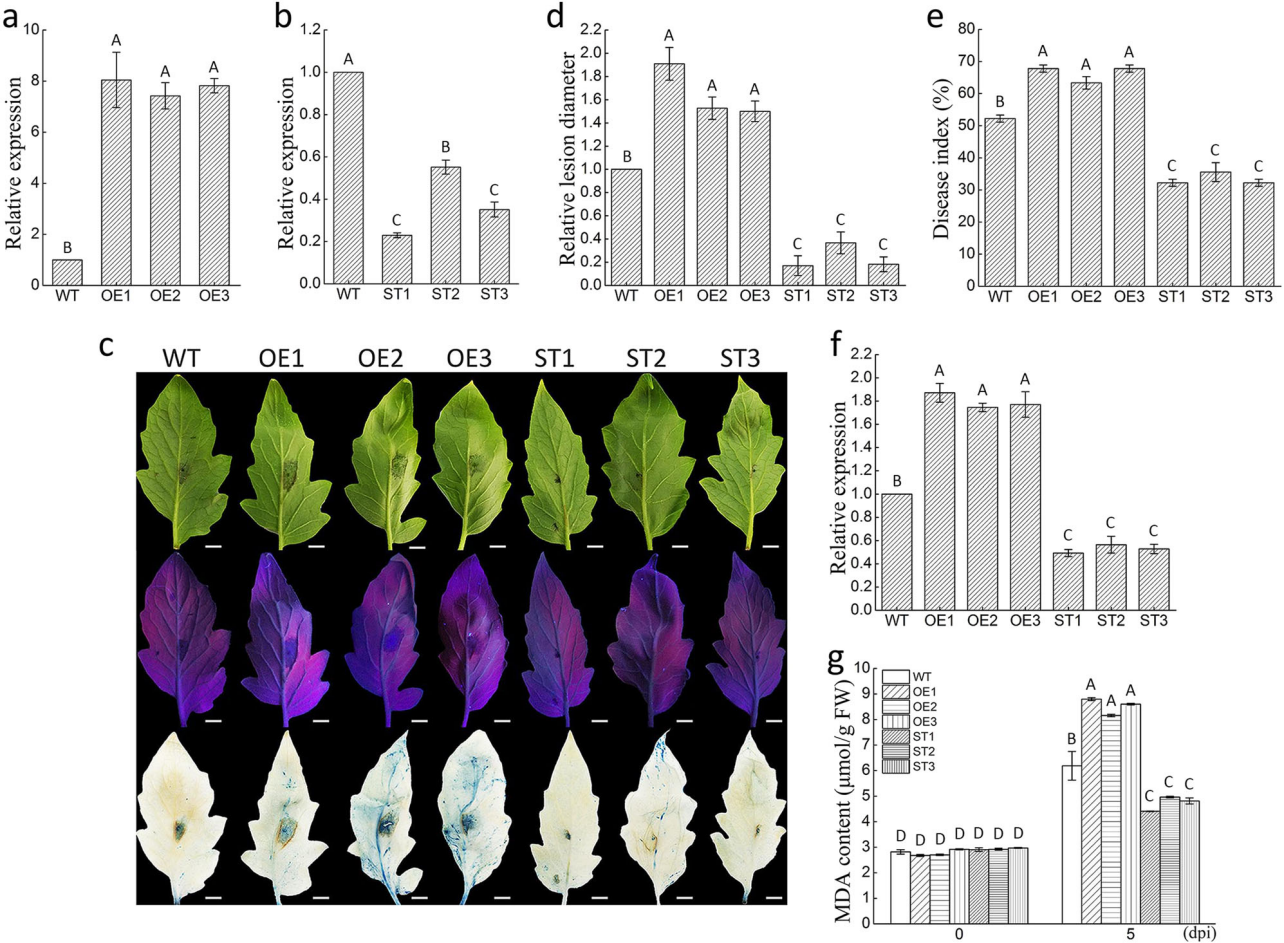
Effect of miR482b overexpression and silencing on the resistance of transgenic tomato to *P*. *infestans*. **a**,** b** Quantitative real-time PCR analysis of the abundance of miR482b in WT, overexpressing (**a**) and silencing (**b**) tomato lines. **c** Detached-leaf inoculated with *P*. *infestans* showing disease signs (scale bars = 0.5 cm). Top: disease symptoms; middle: leaves were photographed in UV-light; bottom: trypan Blue staining for detection of dead cells. **d** The relative lesion diameter. **e** Whole-plant assay of *P*. *infestans*-infected tomato plants showing disease index of WT, overexpressing and silencing tomato lines. **f** Transcript accumulation o*f P*. *infestans Actin* gene in these inoculated plants. **g** MDA content of the leaves from WT, and overexpressing and silencing tomato lines at 5 days after inoculation with *P*. *infestans*. Data are the means ± SEs from three independent experiments. Letters indicate significant difference among samples, and letters shared in common between or among the groups indicate no significant difference (*P* < 0.05)

The transgenic tomatoes were constructed using pBI121-miR482b and pBI121-STTM482, respectively. Four-week-old tomato seedlings (WT and representative positive transgenic lines) were transferred to pots and grown for 2 weeks before they were subjected to qRT-PCR analysis to estimate the abundance of miR482b in their leaf tissue. MiR482b was more abundant in all overexpressing lines and less abundant in all silencing lines compared to WT lines (Fig. 3a, b) with the expression in three overexpressing lines (OE1, OE2, and OE3) being ~8-fold, 7.4-fold, and 7.8-fold, respectively, the expression in WT, while in three silencing lines (ST1, ST2, and ST3) the expression were 0.32-fold, 0.28-fold, 0.36-fold, respectively, the expression in WT. In addition, after genomic DNA-PCR detection based on a pair of specific primers of *nptII*, transgenic lines exhibited the expected transgene-specific band (395 bp), WT was not found (Figure S2).

### MiR482b enhances tomato susceptibility to *P*. *infestans*

After the inoculation of *P*. *infestans*, the detached leaves were gradually darkened and the disease symptoms gradually appeared. Compared to WT, the disease symptoms were more severe on the leaves of miR482b-overexpressing lines and less severe on the leaves of miR482b-silencing lines (Fig. 3c). The dead cells of the treated leaves were examined by trypan blue staining. The darker blue marks in the leaves of overexpressing plants indicated that a greater number of dead cells occurred in miR482b-overexpressing leaves than WT, while the dark blue marks were reduced after miR482b silenced which demonstrated fewer dead cells were existed in miR482b-silencing leaves compared to WT (Fig. 3c). Furthermore, lesion diameters of miR482b-overexpressing and miR482b-silencing leaves were significantly longer and shorter than WT leaves, respectively (Fig. 3d).

In the whole-plant inoculation assay, the DI was found to be higher in miR482b-overexpressing lines than WT, which indicated that blight lesions obviously occupied more leaf area in tomato plants overexpressed miR482b. Conversely, compared to WT, the DI of silencing lines was significantly reduced (Fig. 3e). The transcript levels of *P*. *infestans Actin* gene measured by qRT-PCR were used to indicate *P*. *infestans* growth in plant and the result showed a significant increase and decrease in abundance of *P*. *infestans* in overexpressing and silencing lines compared to WT, respectively (Fig. 3f). In plant–pathogen interaction, plant cellular membranes were damaged because of late massive ROS generation^56^. MDA, which is taken as an indicator of membrane damage^50^, was detected in the overexpressing, silencing and WT plants inoculated with *P*. *infestans*. A significantly higher MDA level was found in overexpressing lines, suggesting that these plants suffered more severe membrane damage than WT (Fig. 3g). In silencing lines, the lower MDA level suggested that they suffer less severe membrane damage compared to WT plants (Fig. 3g). To some extent, these results also suggested that miR482b had led to the enhancement of tomato susceptibility to *P*. *infestans*.

### Identification of miR482b targets

**Fig. 4:**
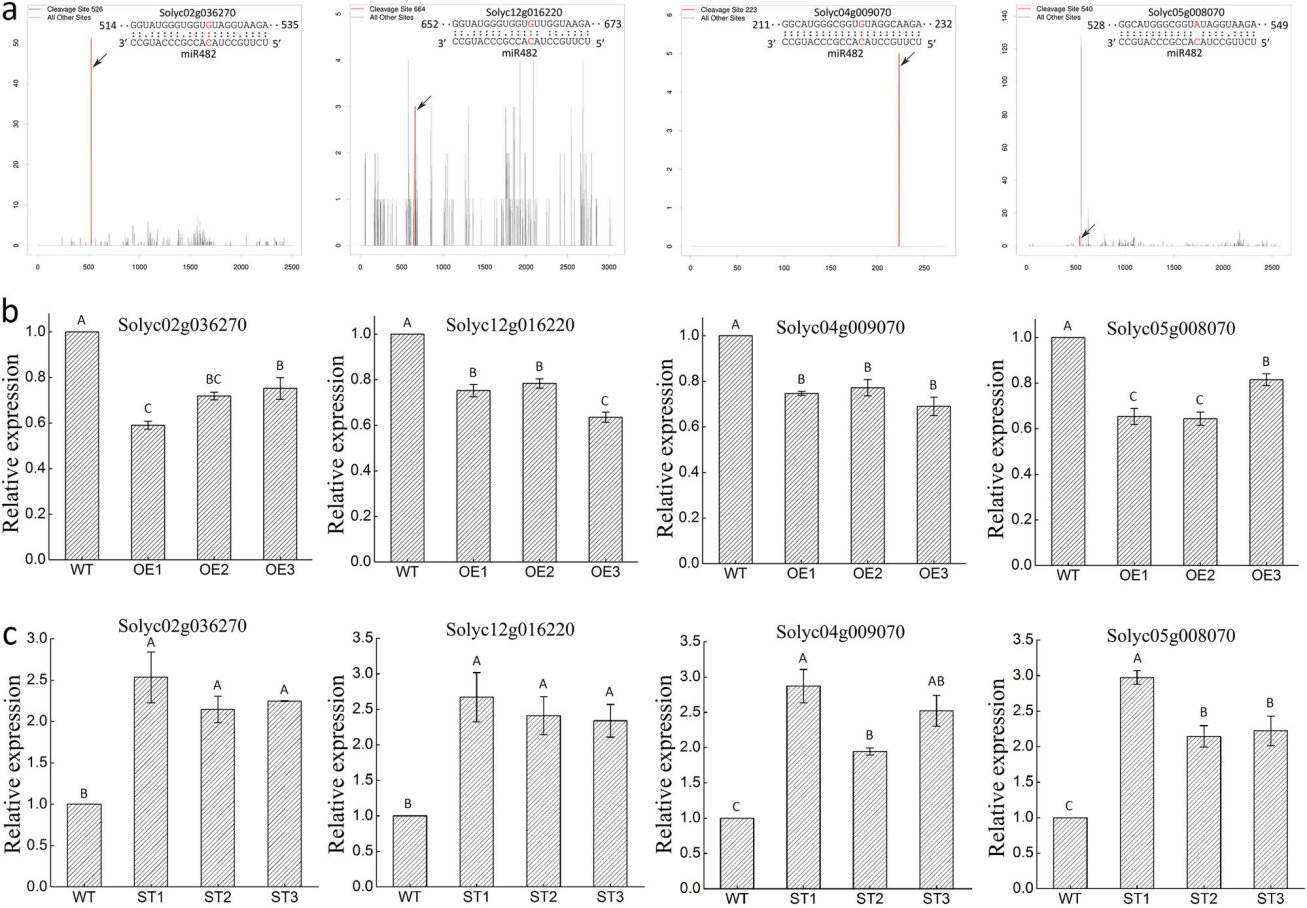
Identification of miR482b target genes in tomato. **a** T-plots showing the miR482b cleavage sites of target genes, *Solyc02g036270*.*2*, *Solyc12g016220*.*2*, *Solyc04009070*.*1*, and* Solyc05g008070*.*2*. The bar graph shows the number of sRNA reads mapped to each position on the transcript (*x* axis) and the sRNA raw read number (*y* axis). Arrow indicates cleavage sites. **b**, **c** Quantitative real-time PCR analysis of the expression levels of target genes in WT and the transgenic tomato overexpressed (**b**) and silenced (**c**) miR482b. Data are the means ± SEs of three independent experiments. Various letters indicate significant difference among samples, and letters shared in common between or among the groups indicate no significant difference (*P* < 0.05)

Identification of target genes is essential to show the regulatory networks of miRNA. Prediction of miR482b target genes using psRNATarget and WMD3 showed 30 *NBS–LRR* disease-resistance genes (Table S1). Four of these genes, *Solyc02g036270*.*2*, *Solyc04g009070*.*1*, *Solyc12g016220*.*2*, and *Solyc05g008070*.*2*, were found to have been cleaved at the miR482b-specific cleavage site according to the available tomato degradome sequencing data, indicating that they were the target genes of miR482b (Fig. 4a). Overexpression of miR482b led to significant reduction in the expression of its four target genes (Fig. 4b). On the contrary, these four target genes was upregulatedly expressed in miR482b-silencing tomatoes (Fig. 4c).

**Fig. 5:**
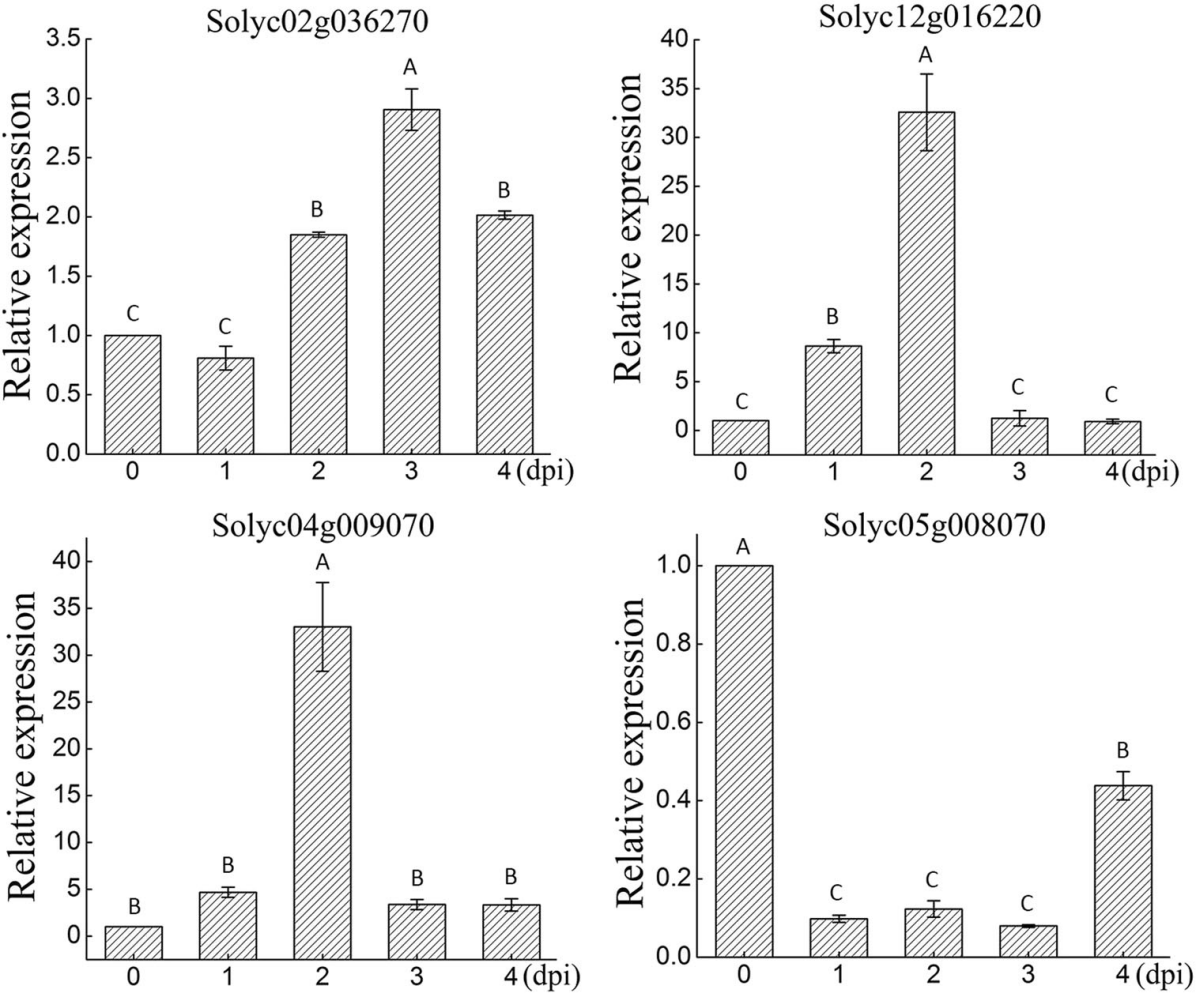
Expression patterns of target genes in tomato leaves at the indicated time after being sprayed with *P. infestans*. Data are the means ± SEs of three independent experiments. Various letters indicate significant difference among samples, and letters shared in common between or among the groups indicate no significant difference (*P* < 0.05)

Upon infection by *P*. *infestans*, the expression levels of *Solyc02g036270*.*2*, *Solyc04g009070*.*1*, and *Solyc12g016220*.*2* were initially increased, but then decreased (Fig. 5). Their expression trend was in contrast to that of miR482b, which further verified the relationship between miR482b and the three genes. However, the expression level of *Solyc05g008070*.*2* was decreased at the first day after infection by *P*. *infestans*, but remained constant for the next two days, and then increased (Fig. 5), suggesting a lack of correlation with the expression trend of miR482b. According to the tomato degradome sequencing data, *Solyc05g008070*.*2* was also found to have been cleaved by miR6024, which might be the reason why there was no correlation between the expression of miR482b and *Solyc05g008070*.*2*. These results therefore indicated that miR482b might play an important role in the regulation of *NBS–LRR* genes during tomato–*P*. *infestans* interaction.

## Discussion

To gain insight into the molecular mechanisms responsible for the late blight resistance, we previously used high-throughput sequencing to identify *P*. *infestans*-induced miRNAs in tomato, including miR169, miR398, miR482, miR6024, miR6026, and miR6027^25,47,48^. Among these miRNA candidates, miR482 is one of the most important miRNAs involved in plant–pathogen interaction. In this study, we showed that the abundance of miR482b in tomato was reduced following *P*. *infestans* infection (Fig. 1b). Similar results have also been reported by other investigators, who found that decreased abundance of miR482 occurs in *Solanaceae* upon infection with different pathogens, including *V*. *dahlia*, *Fusarium oxysporum*, etc.^30,31^. Recently, it was found that RNA silencing suppressors from pathogens might suppress the accumulation of miRNA in plant–pathogen interaction^57^. For example, two effectors form the soybean pathogen *P*. *sojae*, named *Ps*PSR1 and *Ps*PSR2 suppressed transgene silencing by inhibiting the accumulation of plant sRNAs^58,59^. In addition, the accumulation of miRNA also was regulated by lncRNAs and transcription factors. For example, in tomato–TYLCV interaction, a tomato lncRNA, slylnc0195 acted as a target mimic to suppress the expression of miR166a^60^. Meanwhile, ripening inhibitor (RIN), a vital transcription factor acted though sequence-specific interactions with cis-regulatory DNA elements in the promoter of MIR172a to regulate its accumulation^61^.

Tomato L3708 is a resistant tomato to *P*. *infestans*. But, the miR482b sequences in tomato L3708 and cultivated tomato showed 100% sequence identity (Figure S1). To study the function of miR482b in tomato–*P*. *infestans* interaction, we introduced miR482b into cultivated tomato to constructed transgenic tomato. Three independent transgenic tomato lines were generated, which increased transcript amounts of miR482b. After the infection of *P*. *infestans*, more serious disease symptoms occurred in tomatoes overexpressed miR482b than WT, which suggested miR482b could enhance the susceptibility of tomato to *P*. *infestans* infection (Fig. 3). Similar results have also been reported in potato. The overexpression of miR482e in potato led plants to be sensitive to *V*. *dahliae* infection^31^. These results implicate a role for miR482b in the defense response of tomato against *P*. *infestans* infection.

As overexpression is not enough to fully understand miRNA functions and some consequence from overexpression studies may be caused by temporal or spatial misexpression^62^, miRNA-silencing technology should be used to explore functions of miRNAs. STTM, an effective tools to restrain miRNA activity, make it technically possible to silence miRNAs in various plants. In rice, transgenic STTM lines silencing 35 miRNA families (miR398, miR172, miR156, etc.) were produced as resources for functional studies and crop improvement^62^. STTM was also be used in tomato to silence miR396, which resulted in tomato flowers, sepals and fruits all obviously becoming bigger^63^. Meanwhile, effectively inhibiting the expression of miR858 by STTM induced anthocyanin accumulation in tomato^22^. In present study, we used STTM to silence miR482b to validate its function. In miR482-silencing lines, with the abundance of miR482b reduced significantly, plant resistance to *P*. *infestans* enhanced observably, which further proved miR482b could enhance the susceptibility of tomato to *P*. *infestans*.

In this study, four *NBS–LRR* genes were identified as the target genes of miR482b by degradome sequencing and qRT-PCR (Fig. 4). All these target genes showed more than 95% sequence identity and their binding regions of miR482b are completely identical between tomato L3708 and cultivated tomato. For example, the sequence identity of the target gene, *Solyc02g036270*.*2* is 99% in these two tomato source (unpublished). That is to say, miR482b cleaves the same miR482b-specific cleavage site in tomato L3708 and cultivated tomato. NBS–LRR proteins have been shown to function as immune receptors that recognize specific pathogen effector proteins during plant–pathogen interaction, leading to the initiation of plant ETI^25,28,64^. The simplest model for its functioning is that NBS–LRR proteins recognize their corresponding effectors from various pathogens, and their interaction would lead to the activation of a series of defense responses^65^. Before the attack of a pathogen, the expression level of *NBS–LRR* genes is very low, but would rapidly increase at the stage of infection^66–68^. For example, *VaRGA1*, a grapevine *NBS–LRR* gene, is strongly induced by *Plasmopara viticola* at 12 hpi^65^. The expression of a novel peanut *NBS–LRR* gene, *AhRRS5*, is also increased in response to *R*. *solanacearum* infection, both in resistant and susceptible peanut cultivars^69^. Similar to these previous studies, the expression of *Solyc02g036270*.*2*, *Solyc04g009070*.*1*, and *Solyc12g016220*.*2*, three of the four *NBS–LRR*s genes identified as target genes of miR482b, were also elevated after *P*. *infestans* infection, which clearly demonstrated that these genes could respond to the infection of *P*. *infestans* in tomato (Fig. 5). In addition, previous studies also showed that overexpression of the *NBS–LRR* gene *VaRGA1* in *Nicotiana benthamiana* would enhance its resistance to *P*. *parasitica*^65^, whereas silencing of a *CC–NBS–LRR* gene through VIGS in cotton dramatically increased its susceptibility to *V*. *dahliae*^70^. Similar results were obtained in this study. The suppression of *NBS–LRRs*, which was caused by the overexpression of miR482b (Fig. 4b), led to more disease symptoms in miR482b-overexpressing tomato lines (Fig. 3). In miR482b-silencing lines, the expression levels of *NBS–LRRs* was significantly upregulated (Fig. 4c) and the resistance of tomato plants to *P*. *infestans* enhanced (Fig. 3). Thus, these results provided additional evidence that these *NBS–LRR* genes were targeted by miR482b and they might play a role in the activation of tomato defense responses.

**Fig. 6:**
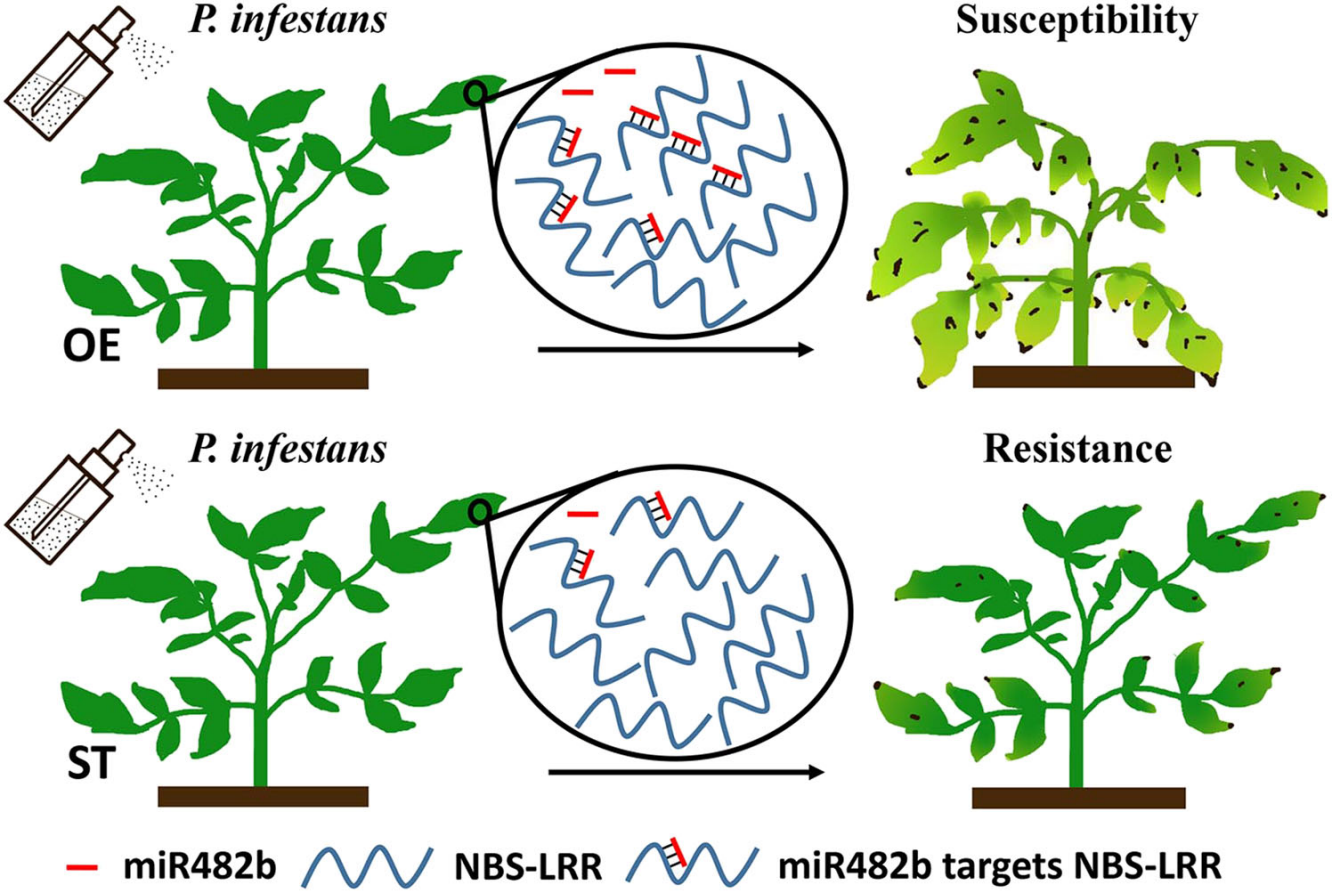
Model of miR482b-NBS-LRR regulation network involved in the tomato–*P. infestans* interaction. Overexpression of miR482b in tomato negatively modulated *P*. *infestans* defense response by decreasing the abundance of NBS–LRRs, while the resistance was enhanced after *miR482b* silencing

Based on these results, we propose a working model to explain how miR482b affects tomato resistance to *P*. *infestans* (Fig. 6). Following our results for degradome analysis, miR482b cleaves its target genes, the members of *NBS–LRR* family to suppress the abundance of NBS–LRRs (Fig. 4). When miR482b is overexpressed in tomato, the abundance of NBS–LRRs is decreased and more serious disease symptoms are shown after *P*. *infestans* infection. On the contrary, in miR482b-silencing tomato plants, more abundance of NBS–LRRs results in enhancement of tomato resistance to *P*. *infestans*. Our results provide insight into tomato miR482b–*NBS–LRR* module involved in the response to *P*. *infestans* infection and guidance for molecular breeding to improve *P*. *infestans* stress tolerance in the future.

## Electronic supplementary material

Figure S1

Figure S2

Table S1

Table S2
