# Supplementary figures and images for: Function identification of miR482b, a negative regulator during tomato resistance to Phytophthora infestans
Source: Hortic Res. 2018 Mar 1;5:9. doi: 10.1038/s41438-018-0017-2 (PMC5830410; doi:10.1038/s41438-018-0017-2)

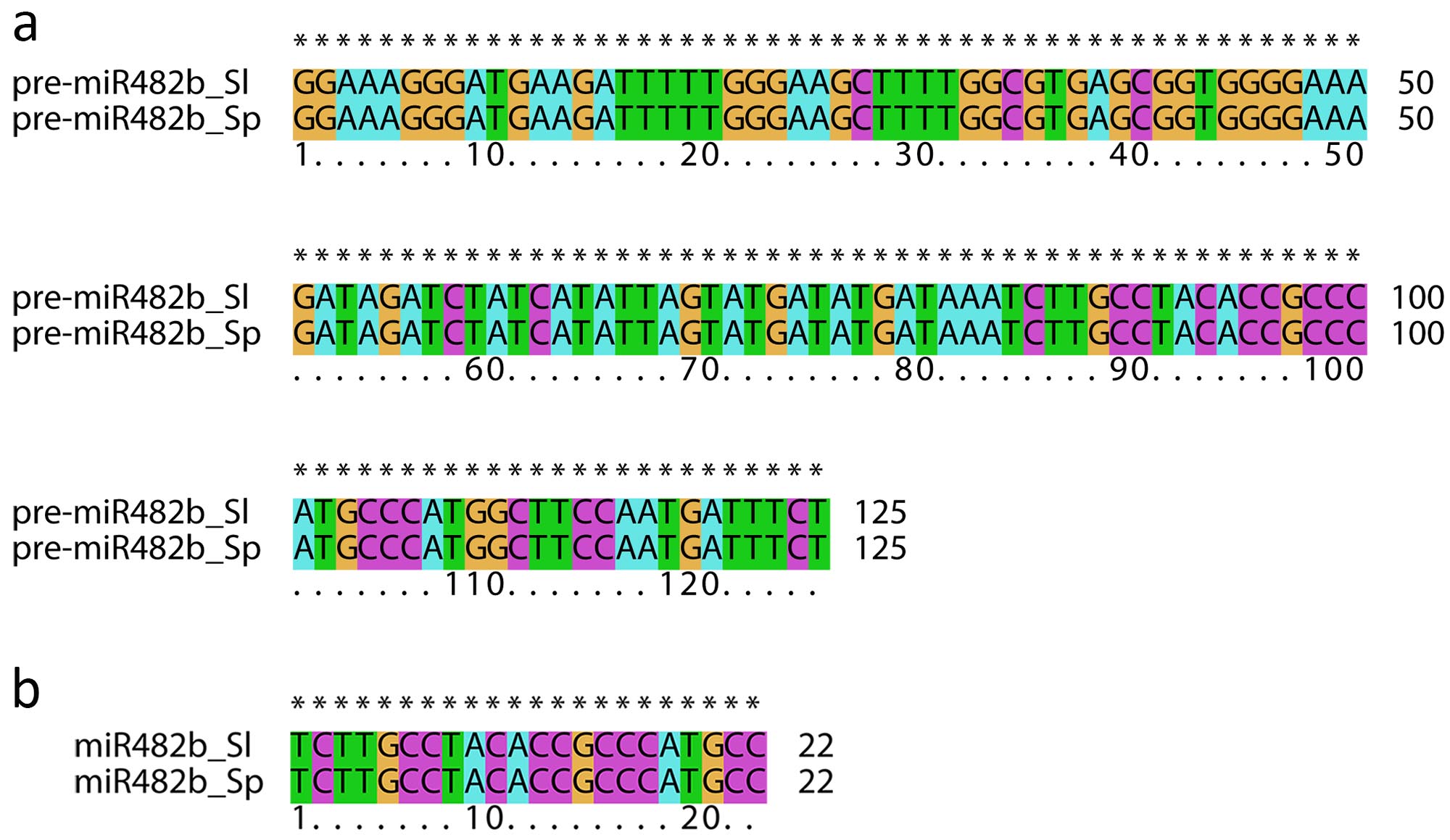

Supplement: Supplementary file 1 — Figure S1 [file 41438_2018_17_MOESM1_ESM.jpg]

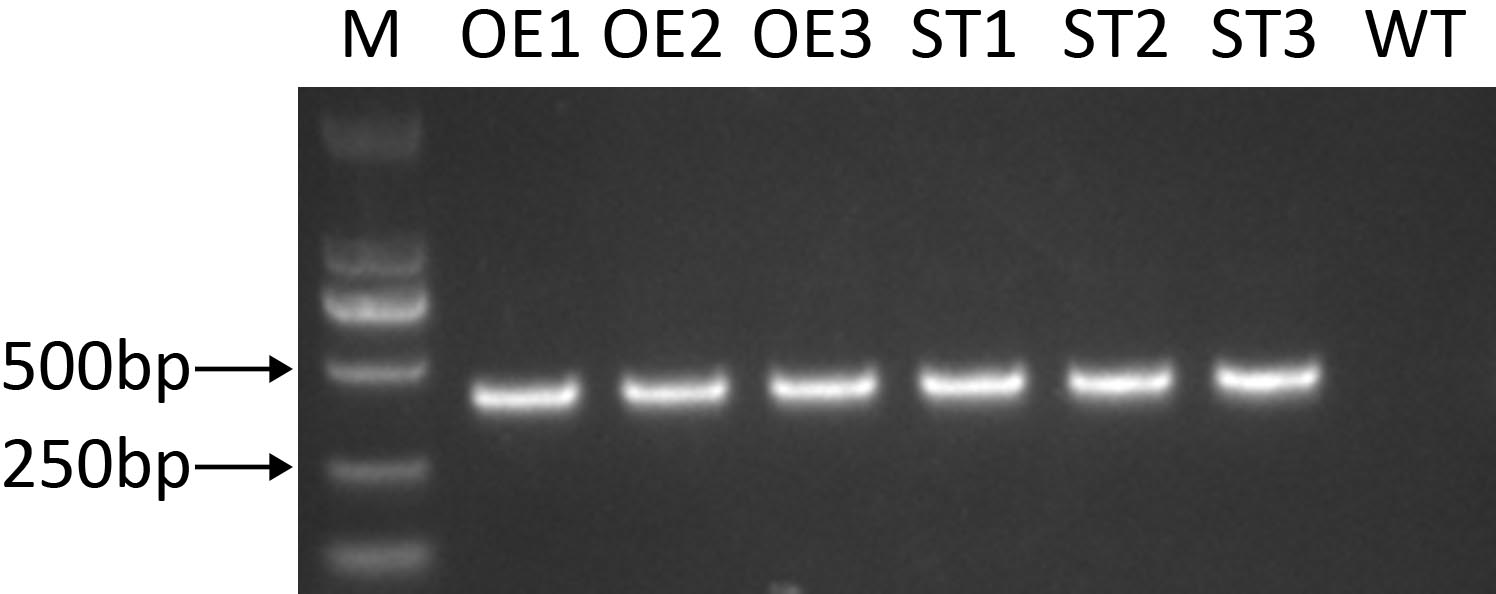

Supplement: Supplementary file 2 — Figure S2 [file 41438_2018_17_MOESM2_ESM.jpg]
