# Supplementary material for: Function identification of miR482b, a negative regulator during tomato resistance to Phytophthora infestans
Source: Hortic Res. 2018 Mar 1;5:9. doi: 10.1038/s41438-018-0017-2 (PMC5830410; doi:10.1038/s41438-018-0017-2)
Supplement: Supplementary file 3 — Table S1 [file 41438_2018_17_MOESM3_ESM.docx]

| **miRNA Acc.** | **Target Acc.** | **Target_start** | **Target_end** |
| --- | --- | --- | --- |
| miR482b | Solyc02g036270.2.1 | 514 | 535 |
| miR482b | Solyc05g008070.2.1 | 528 | 549 |
| miR482b | Solyc04g009120.1.1 | 601 | 622 |
| miR482b | Solyc12g017800.1.1 | 526 | 547 |
| miR482b | Solyc11g065780.1.1 | 454 | 475 |
| miR482b | Solyc04g025820.1.1 | 314 | 335 |
| miR482b | Solyc07g039420.1.1 | 568 | 589 |
| miR482b | Solyc04g025840.1.1 | 370 | 391 |
| miR482b | Solyc12g016220.1.1 | 652 | 673 |
| miR482b | Solyc10g054940.1.1 | 46 | 67 |
| miR482b | Solyc10g055170.1.1 | 46 | 67 |
| miR482b | Solyc11g006530.1.1 | 526 | 547 |
| miR482b | Solyc11g006630.1.1 | 532 | 553 |
| miR482b | Solyc04g009290.1.1 | 574 | 595 |
| miR482b | Solyc04g009130.2.1 | 597 | 618 |
| miR482b | Solyc07g039400.1.1 | 337 | 358 |
| miR482b | Solyc10g054970.1.1 | 539 | 559 |
| miR482b | Solyc07g005770.2.1 | 541 | 562 |
| miR482b | Solyc11g071420.1.1 | 622 | 643 |
| miR482b | Solyc08g005440.2.1 | 616 | 637 |
| miR482b | Solyc11g020100.1.1 | 520 | 541 |
| miR482b | Solyc11g020090.1.1 | 103 | 124 |
| miR482b | Solyc04g009070.1.1 | 211 | 232 |
| miR482b | Solyc04g009110.1.1 | 574 | 595 |
| miR482b | Solyc04g009240.1.1 | 565 | 586 |
| miR482b | Solyc04g009250.1.1 | 577 | 598 |
| miR482b | Solyc04g009260.1.1 | 580 | 601 |
| miR482b | Solyc04g009270.2.1 | 440 | 461 |
| miR482b | Solyc04g009660.2.1 | 550 | 571 |
| miR482b | Solyc04g009690.1.1 | 520 | 541 |

**Table S1**  Target genes of *Sp*miR482b predicted by psRNATarget and WMD3
