# Supplementary material for: Function identification of miR482b, a negative regulator during tomato resistance to Phytophthora infestans
Source: Hortic Res. 2018 Mar 1;5:9. doi: 10.1038/s41438-018-0017-2 (PMC5830410; doi:10.1038/s41438-018-0017-2)
Supplement: Supplementary file 4 — Table S2 [file 41438_2018_17_MOESM4_ESM.docx]

**Table S2**  Primers used in this study

| **Name** | **Primer Sequence** | **Function** |
| --- | --- | --- |
| c-miR482F | CGGGATCCAAAATTTGTAAGAGTGGTGCATGA | Pre-miR482b Cloning |
| c-miR482R | CGAGCTCTGTACTCTATTTGTCGCTGATTTTG |  |
| q-miR482 | TCTTGCCAATACCGCCCAT | miR482b qRT-PCR |
| Solyc02g036270.2.1F | GGTATGGGTGGTGTAGGTAAGA | Target gene qRT-PCR |
| Solyc02g036270.2.1R | GACTGACAGTGACCATGACAAC |  |
| Solyc05g008070.2.1F | TTATTGGCATGGGCGGTATAG | Target gene qRT-PCR |
| Solyc05g008070.2.1R | GACCCATGCATGTTTCTCAAATC |  |
| Solyc12g016220.1.1F | TATGTGGTATGGGTGGTGTTG | Target gene qRT-PCR |
| Solyc12g016220.1.1R | TAAGGTCTGGTTGTTGACTGAC |  |
| Solyc04g009070.1.1F | TCTCAAACCAGAGCCTTGTC | Target gene qRT-PCR |
| Solyc04g009070.1.1R | CTAGGACTTCTGTAGAGGTTTCTC |  |
| nptIIF | TGCCCTGAATGAACTGCAGG | Identification of transgenic tomato |
| nptIIR | TCACGACGAGATCATCGCCG |  |
| Pi ActinF* | GTTCCTGAGTTTTTGCTCCATC | Pi abundance |
| Pi ActinR | GCAGACCCTTTGCTACTACCTT |  |
| Tomato ActinF | TGTGTTGGACTCTGGTGATGGTGT | Reference |
| Tomato ActinR | ATCCAAACGAAGAATGGCATGCGG |  |

*Pi, *Phytophthora infestans*
